# A Letter

**Published:** 1877-08-20

**Authors:** 


A LETTER from Dr. T. B. Swift, a medical friend of Cooksville, Miss., thus concludes. We hope his inquiries will be answered:

"We would be pleased to see something from some brother M.D. on the patholigy and treatment of some diseases that seem to be prevailing through portions of our Southern country, to-wit: Hæmorrhagic Malarial Fever, or yellow chills; Typhoid Pneumonia; Pernicious Fever; Puerperal Peritonitis, and Ante et Post Partum Eclampsia; Hydro-pericarditis, and Endo-pericarditis; Hydrothorax. Hoping to hear from some brother physician on the above maladies, with respect I remain yours, etc."

